# The functionality of unit practice councils and its relationship with nurses' accountability: A cross‐sectional study

**DOI:** 10.1002/nop2.1722

**Published:** 2023-03-20

**Authors:** Mohammad Abu Dawass, Huthaifah Khrais, Ahmad Rayan, Mohammad Jaber

**Affiliations:** ^1^ Ped Emergency King Fahad Medical City Riyadh Saudi Arabia; ^2^ Faculty of Nursing Zarqa University Zarqa Jordan

**Keywords:** Accountability, nurse, Saudi Arabia, shared‐governance, unit practice councils

## Abstract

**Aim:**

This study aimed to examine the relationship between perceived unit practice councils' functionality and nurses' accountability in Saudi Arabia.

**Design:**

A descriptive, correlational and cross‐sectional design was used.

**Methods:**

Convenience sampling of 160 nurses working in multiple sites of a large, tertiary medical centre in Saudi Arabia was performed. Structured self‐administered questionnaires were used to measure perceived unit practice council functionality and levels of nurse accountability.

**Results:**

Nurses perceived that unit practice councils had moderate levels of functionality; however, nurses had high levels of accountability. Also, perceived unit practice councils' functionality had a significant positive relationship with nurses' accountability (*r* = 0.49, *p* < 0.001). More studies are needed to investigate the impact of different shared governance models on nurses' work environments.

## 1 INTRODUCTION

A positive and healthy work environment is considered an essential and crucial component to increase nurses' satisfaction; therefore, nurses used shared governance and its related Unit Practice Councils (UPCs) as a way to attain it. UPCs are innovative management and leadership process built on shared decision‐making among all nurses and focused on nurses' practices and accountability of their care (Lamoureux et al., 2014).

Engaging nurses at the service point in raising clinical and operational issues is linked to greater levels of empowerment and confidence pertaining to shared governance and its related UPCs (Al‐Marri & Kehyayan, 2018; Brull, 2015). Implementation of professional governance structures can enhance the synergistic work environment between the management and frontline employees, and improve employee work conditions enhancing teamwork, open‐mindedness and the adventure of finding new solutions to old problems (Fuentes et al., 2019).

One of the ways to implement shared governance is through the creation and institutionalization of UPCs which are groups of nurses within units who take lead in implementing, monitoring, evaluating standard and consistent practice among staff nurses and the wider multidisciplinary team (Sloan, 2020). UPCs should be well‐structured and functional, with nurses aligning themselves with self‐directed work teams and organizational leaders' competencies (Joseph & Bogue, 2016). Optimal functioning of UPCs has been shown to result in nursing empowerment, improved clinical practice, a healthy work environment, improved peer relationships, cultural change, improved perceptions of patients and employees, and improved patient outcomes and satisfaction (Gerard et al., 2016; Wessel, 2012). However, a significant challenge in the research of UPCs is measuring its functionality as several healthcare organizations create UPCs that fit local demands, governance requirements, and patient needs (Fray, 2011).

On the other hand, accountability is a hallmark of the nursing profession. Nursing organizations around the world demand that nurses demonstrate accountability in their daily practice as part of professional and ethical standards. When issues on patient safety and quality of nursing care arise, nurses are expected to be both accountable and responsible for their actions. However, as of yet, there is no standard or consensus definition of accountability (Krautscheid, 2014). Some authors define accountability as a multidimensional concept that pertains to professional expectations of taking responsibility for actions when things go wrong, as societal expectations of performing actions within the boundaries of what society views as acceptable behaviour, as transparency of actions when nurses decide to perform or carry out a task, as outcome‐based attitude of evaluating actions according to the value of results (e.g. positive or negative, beneficial or detrimental, etc.), and as strategic‐oriented attitude of performing actions with the intent of producing output in the most efficient manner that is based on goals and objectives (Leonenko & Drach‐Zahavy, 2016; Wandersman et al., 2016). In consequence, the lack of a standard definition creates problems for nurse researchers and practitioners in the use of accountability as a concept, in measuring the extent and substance of accountability, and in teaching and learning accountability as a professional trait (Krautscheid, 2014).

However, there is a dearth of studies that examined the effectiveness of unit practice councils as a shared governance framework and the relationship between UPCs and nurse accountability resulting in a gap in knowledge on whether or not the implementation of shared governance via UPCs improves the accountability of nurses on the bedside. Studies have shown that when nurses are engaged and empowered to participate in the decision‐making processes of the healthcare organization, they are less likely to report poor outcomes on job performance, job satisfaction, patient care, patient safety, and nursing care quality (Abu Mansour & Abu Shosha, 2022; Alrwaihi et al., 2018). Also, it has been suggested that specific issues regarding nurses' accountability are needed to be linked with UPCs, that is, nurses are able to make accountable decisions at different levels. For example, at the clinical‐practice level, nurses are held accountable for making decisions regarding nursing care delivery models, quality of care, and performance appraisals. However, at the management‐practice level, nurses must participate in managing resources and designing organizations (Moreno et al., 2018).

Results of the study can explicate the role of shared governance on the extent and substance of nurse accountability at the individual and organizational levels, and aid nurse managers, hospital administrators and nurse leaders in creating UPCs whose structures and frameworks have the potential to promote nurse empowerment, enhance nurse capacities, ensure standardization and consistency of practices and promote patient safety and quality of healthcare services (Gerard et al., 2016).

In Saudi Arabia, the exact relationship between UPCs and accountability has not been well addressed in previous literature; therefore, we aim to study the relationship between UPCs and nurses' accountability. The following research questions were specifically addressed:

- What are the levels of perceived UPCs functionality and accountability among Saudi Arabian nurses?
- Is there a relationship between UPCs functionality and Saudi Arabian nurses' accountability?

## 2 METHODS

### 2.1 Research design

The study utilized a descriptive, cross‐sectional, correlational design.

### 2.2 Setting and sampling

The study was performed in multiple sites belonging to a large, tertiary medical complex in Saudi Arabia. The medical complex is made up of four hospitals and seven centres with a total of approximately 1200 beds. Shared governance frameworks were instituted in 2017 via the implementation of unit practice councils. Currently, there are seven UPCs in the medical complex namely (1) scheduler council, (2) heroes' council, (3) informatics council, (4) quality council, (5) educational council, (6) operational council and (7) disaster council.

A convenient sampling technique was used to select nurses with the following inclusion criteria: (1) having at least a bachelor's degree in nursing; (2) having a full‐time contract; (3) having at least 1 year of clinical experience; (4) providing direct patient care; (5) willing to participate in the study. However, nurse managers from top management positions were excluded from the study. In total, 160 nurses returned and completed the questionnaires appropriately.

### 2.3 Instruments

#### 2.3.1 Perceived UPCs functionality

The tool was developed by Ojeda et al. (2014) in a study assessing the role of UPCs in improving nursing work environments in the United States. The tool consists of 16 items of a 5‐point Likert scale valued numerically from 1–5 points wherein: ‘Don't Know = 1’, ‘Strongly Disagree = 2’, ‘Disagree = 3’, ‘Agree = 4’, ‘Strongly Agree = 5’. The potential range of total scores ranges from scores 16 to 80. The scale is valid and reliable with Cronbach *α* of 0.74 (Ojeda et al., 2014).

#### 2.3.2 Accountability index tool

The tool was developed by Specht and Ramler (1994), the tool is composed of 11 items measuring the perceived accountability of nurses using a four‐point Likert scale from ‘Strongly Disagree = 1’ to ‘Strongly Agree = 4’. The construct validity of the scale has been confirmed by a previous study (Drach‐Zahavy et al., 2018). Also, in this study, Cronbach's *α* for the total score was 0.88.

### 2.4 Ethical consideration and data collection procedures

The study was reviewed and approved by the Institutional Review Board (IRB) of ‘REDACTED’ and ‘REDACTED’. Participation in the study was voluntary, an online informed consent was sought from participants prior to data collection, and the participants provided the consent by agreeing to proceed to the survey. Due to the COVID‐19 restrictions, data were collected online between February 2021 and May 2021. Google Form was created and the link to the form was shared with nurse managers in each department, and then, they forwarded that link with nurses.

### 2.5 Data analysis

Statistical analyses were performed using SPSS Software version 24 (McCormick & Salcedo, 2017). Descriptive analysis was used to summarize sample characteristics. Inferential analyses were used to test the relationship between UPCs and nurses' accountability.

Descriptive and inferential statistics were performed to analyse the data. The Statistical Package for the Social Sciences (SPSS) Version 24 was used. Means and standard deviations, wherever applicable, were calculated for the demographic characteristics of the participants. Pearson's *r* was calculated to determine any relationship between the variables. Internal consistency reliability was evaluated using Cronbach's *α* for each of the scales used for data collection in this study. A significance level of *p* < 0.05 was set a priori for all analyses.

## 3 RESULTS

### 3.1 Nurses' background variables

The study consisted of 160 participants. The majority of the participants were female (*n* = 111, 66.4%). More than half of the participants were more than 35 years old (*n* = 83, 51.9%). The sample primarily consisted of registered staff nurses (*n* = 111, 69.4%). Almost 84% of the participants had a Bachelor's degree (*n* = 134, 83.8%) while one had a doctorate. In terms of length of experience as a registered nurse, 27 participants (16.9%) had less than 5 years of experience, 34 (21.3%) had 6–10 years of experience, 34 (21.3%) had 11–15 years of experience, and 65 (40.6%) had more than 15 years of experience. Most of the participants had either 1–5 years (*n* = 53, 33.1%) or more than 10 years (*n* = 65, 40.6%) of working experience in the research setting (Table 1).

**TABLE 1:** Nurses' background variables (*n* = 160).

Variable	Frequency (%)
Gender	
Male	49 (30.6)
Female	111 (69.4)
Age	
<25	5 (3.1)
26–30	30 (18.8)
31–35	42 (26.3)
>35	83 (51.8)
Educational attainment	
Bachelor's	134 (83.8)
High diploma	14 (8.8)
PhD	1 (0.6)
Master's	11 (6.8)
Professional title	
Registered nurse	111 (69.4)
Unit manager	13 (8.1)
Senior manager	36 (22.5)
Years of experience as a registered nurse	
<5	27 (16.9)
6–10	34 (21.3)
11–15	34 (21.3)
>15	65 (40.5)
Years of experience working in King Fahd Medical City	
<1	9 (5.6)
1–5	53 (33.1)
6–10	33 (20.6)
>10	65 (40.7)

### 3.2 Nurses' perceptions of UPCs functionality

Unit practice council functionality was measured using the tool by Ojeda et al. (2014). The mean score was 58.3 which meant that nurses perceived a moderate level of UPCs functionality. As presented in Table 2, the highest scoring item pertained to the ability of UPC to empower nurses and influence decision‐making (*M* = 4.86, SD = 1.1), and nurses know the UPC representatives and how to contact them (*M* = 4.35, SD = 1.5). However, the lowest scoring items were knowing when the last UPC meeting was held (*M* = 2.85, SD = 2.1) and giving an example of a UPC initiative that was implemented in their areas of work (*M* = 2.60, SD = 2.0).

**TABLE 2:** Nurses' perceptions of UPCs functionality (*n* = 160).

Item	Mean (SD)
1. I know the name of my UPC chair or representative and how to contact him or her.	4.35 (1.5)
2. The UPC chair or representative communicates with me as a staff member on the activities of the UPC on a regular basis.	3.91 (1.1)
3. The UPC has positively impacted the care of patients in my unit and improved patient outcomes.	3.84 (1.2)
4. The UPC has positively impacted the practice environment in my unit.	3.87 (1.1)
5. The UPC has empowered me to influence decision‐making that affects me, my unit and my patients.	4.86 (1.1)
6. I am interested in knowing the current activities and initiatives of my UPC.	4.13 (0.9)
7. I believe the UPC and its initiatives are fully supported by administration and management.	4.01 (1.0)
8. My UPC chair or representative values my input.	3.89 (1.2)
9. The role and the function of the UPC are clear	3.68 (1.9)
10. You can provide an example of a UPC initiative that has been implemented in your unit.	2.60 (2.0)
11. The UPC has improved patient outcomes on your unit.	3.38 (2.0)
12. The UPC has improved your practice environment on your unit.	3.40 (2.0)
13. The UPC has served to empower staff members in your unit.	3.27 (2.1)
14. You know the date of the last meeting held by your UPC	2.85 (2.1)
15. A UPC member has actively recruited me to join the UPC.	3.47 (1.9)
16. If I wished to serve on the UPC, my manager or supervisor would allow me paid time to participate in UPC‐related meetings and activities.	3.84 (1.2)

### 3.3 Nurses' accountability levels

As shown in Table 3, the mean score of nurses' level of accountability was 36.8 which meant high levels of being accountable. The highest‐scoring item was being accountable to patients (*M* = 3.44, SD = 0.5) while the lowest‐scoring item was holding peers accountable for their actions (*M* = 3.23, SD = 0.7).

**TABLE 3:** Nurses' level of accountability (*n* = 160).

Item	Mean (SD)
1. I am accountable to my peers for the nursing care I deliver.	3.35 (0.7)
2. I hold my peers accountable for the nursing care they deliver.	3.23 (0.7)
3. I am accountable to patients for the care I deliver.	3.44 (0.5)
4. I am accountable to have the patients I care for prepared for discharge.	3.40 (0.6)
5. I am responsible for defining and monitoring standards of care for the patients on the unit.	3.40 (0.5)
6. I am actively involved in defining standards of care for the patients on the unit.	3.31 (0.6)
7. I am actively involved in monitoring standards of care for the patients on the unit.	3.36 (0.6)
8. I am familiar with the standards of care pertaining to my patients and use the standards to guide my practice.	3.34 (0.5)
9. I am accountable for acquiring the knowledge and skill required to care for the patients on this unit.	3.38 (0.5)
10. If a patient or family member has a complaint about the care under my direction, their concerns should be referred to me and I should contact them with a response.	3.29 (0.5)
11. I regularly consult with nurse peers, read current using literature, attend professional conferences, and incorporate new knowledge into my practice.	3.30 (0.6)
Total	3.35 (0.5)

### 3.4 Correlations between study variables

The study examined whether there is a significant relationship between perceived UPC functionality and accountability. Pearson's *r* showed that the two variables had a significant moderately positive relationship with each other (*r* = 0.49, *p* < 0.001) which meant that nurses who perceived a moderate level of UPCs functionality tend to display high levels of accountability.

## 4 DISCUSSION

Results showed that perceived UPCs in the participating hospital had moderate levels of functionality. The moderate level of functionality suggests that there is room to improve the functions of UPCs. In particular, nurses gave a low score on recalling when the last UPC meeting was held. Regular meetings have the potential to provide a constant source of communication and feedback to staff nurses and UPC members. During such sessions, each individual has the opportunity to highlight the specific area of nursing care and service delivery that needs improvement, issues and concerns that can influence patient safety and the quality of care, learning and development points arising from incidents or near‐misses, and concerns and feedback from patients, families and their significant others (Aldawood et al., 2020). Also, there are opportunities during such meetings to emphasize areas where nurses performed well and had a positive impact on patient and family experience while admitted to the hospital (Gooch, 2016; Abu Mansour & Abu Shosha, 2022). Moreover, UPC members have the opportunity to communicate current patient care status, quality improvement projects, and shared governance initiatives. Conversely, without regular meetings that can promote open communication between staff nurses and UPCs, staff nurses fail to escalate any issues or concerns that can be addressed by specific councils and in turn, UPCs fail to recognize existing problems that nurses encounter on the bedside. As a result, UPC functionality is decreased as its ability to recognize, respond and manage patient‐care problems is hindered by way of lack of regular communication.

More importantly, another item that received a low score was identifying a UPC initiative that was implemented in the nurses' respective units. This result can be concerning in that it may mean that nurses either did not receive communication that a UPC initiative was in place and ongoing within their units or that there were no initiatives were implemented at all. If UPC initiatives were implemented but nurses were not aware of them, information dissemination by and within UPCs is faulty; initiatives and other quality improvement projects are of no use to staff nurses if they were not aware of them in the first place. On the contrary, if information channels were properly functioning and, indeed, UPCs in the hospital did not implement any initiatives at all, then it may suggest that UPCs failed in performance and function. While the literature is clear that UPCs as a shared governance framework have the potential to engage and empower nurses towards collaboration, leadership and safe and quality patient care, UPCs are only effective insofar as their ability to conceptualize, design, plan and implement initiatives and projects that can be translated and influential to actual patient care (Giambra et al., 2018; Wong et al., 2013). Actions by UPCs should be felt by nurses and patients as they are the primary stakeholders of UPC functionality.

There are areas of UPC functionality worth commending as nurses managed to demonstrate high scores in these items. The onus is for UPCs to implement strategies that can sustain the measured high scores over time.

First, participants believed that UPCs empower them and help them to make decisions. Decision making is a vital element for a professional and healthy working environment. This finding is supported by the study of Barden et al. (2011), as they found that shared governance provides a context where nurses are involved in decision‐making processes which increased nurses' satisfaction and improved patients' outcomes.

Second, knowing the people who are part of UPCs and knowing how to contact them suggests that there is an existing communication link between staff nurses and UPCs, and UPCs are not detached and alienated from nursing personnel. In her study, Wessel (2012) found that nurses' communications with their peers and managers inspire them to lead change and facilitate the group decision‐making process.

Lastly, participants expressed interest in knowing the initiatives of UPCs. In turn, UPCs should take advantage of such interest by bolstering information dissemination about current and planned projects, inviting staff nurses to monthly discussion fora and meetups, and encouraging participation in projects and initiatives that nurses find most appealing. By making the most of the interests expressed by nurses, UPCs increase their effectiveness in increasing nurse engagement and participation.

As established in the review of literature, accountability is a concept that is widely accepted in the nursing profession but is poorly defined, thereby limiting the number of researches done on the topic (Krautscheid, 2014). Moreover, some studies have shown that nurses tended to link the concept of accountability with self‐incrimination since nurses were expected to take the blame when a mistake happens to a patient, and challenging the status quo of the healthcare organization since nurses are expected to go against the practice of peer and colleagues (Leonenko & Drach‐Zahavy, 2016). Therefore, it is not surprising that the dimension of accountability nurses had the lowest score in was holding their colleagues accountable for the care that they deliver. This is problematic, though, since one of the roles nurses are expected to perform is that of the patient's advocate in terms of keeping the patient safe, preventing harm from reaching the patient, and acting on the patient's best interests (Choi et al., 2014). If nurses were reluctant to point out mistakes or problems that they have with the way their colleagues deliver nursing care, poor performance, worse patient outcomes, poor patient and family satisfaction, adverse events, avoidable deaths, and breaches in patient safety can potentially and negatively proliferate (Vermeir et al., 2015). Several high‐profile cases that resulted in a significant extent of patient harm and volume of avoidable deaths such as that of the Mid Staffordshire Hospital and Furness General Hospital in the UK was borne out of complacency from the nursing staff to call each other when poor practice was observed, and to have key staff and management personnel accountable for their actions (Newdick & Danbury, 2015; Wise, 2015).

Nonetheless, nurses had overall high scores for accountability, especially for dimensions pertaining to personal accountability for their actions in following and implementing standards of care, monitoring standards of care, delivering nursing care to their patients, keeping their knowledge and skills up‐to‐date with current evidence, preparing their patients for discharge, and responding to any complaint arising from the patient and their families. The high scores highlight nurses' acknowledgement of the value of accountability in patient safety, prevention of harm, and quality of service delivery (Rubio‐Navarro et al., 2020). If nurses recognize that they are accountable for the results of their actions, they have the potential to create situations that protect their patients from injury and promote their health and well‐being.

A significant positive moderately strong relationship was found between UPC functionality and accountability, suggesting that nurses working with UPCs with high functionality tend to demonstrate higher levels of accountability. The relationship implies that a functional UPC has the potential to positively impact the way nurses take accountability for their actions, especially since the core of UPCs is the promotion of safe and quality patient care through empowerment, engagement and participation. In turn, high levels of accountability among nurses' feedback to the UPCs, providing information on areas of service delivery that require improvement and areas that are already being performed to standard and will only need to be sustained.

### 4.1 Implications and recommendations

As discussed, the significance of accountability in practice cannot be overstated. The need to be accountable acts as a safeguard for patients because nurses bear in mind that all their actions in providing nursing care require a rationale that is rooted in evidence, and that mistakes and poor performance have critical ramifications for their ability to practice the profession in the future. Likewise, UPC functionality seems to positively influence the level of nurse accountability, which means that UPCs need to be able to function and perform within expectations and that initiatives and projects need to be felt by patients and staff nurses. More importantly, results are significant in that they have the potential to directly affect actual bedside practice, with the overall goals of both UPC functionality and nurse accountability being the promotion of patient safety, achievement of target patient outcomes, and better workforce well‐being.

The results of the study emphasize the importance of teaching accountability at an early stage of nurses' undergraduate education. This attitude is critical in ensuring the integrity of care received by patients and the quality of service provided by nurses. In addition, the study has laid the groundwork for examining UPC functionality and its links with nurse accountability. With the scarcity of high‐quality evidence on both variables, future studies can look closer at the dimensions that compose UPC functionality and nurse accountability and identify factors that may predict and influence its measurement.

In view of the findings of the study, the following recommendations are made:

- Develop programs and strategies that will target the improvement of UPC functionality. Existing UPCs need to be strengthened, especially in terms of keeping regular communication with staff nurses. In addition, UPC members may require training in the conceptualization, formulation, design, implementation and evaluation of quality improvement projects and evidence‐based initiatives that can be easily identified and practically utilized in reality.
- Create opportunities for staff nurses to engage and participate in UPC activities. These can be in the form of workshops, brainstorming, club sessions and meetups.
- Perform regular evaluation of UPC functionality. Consistent measurement and follow‐up will allow healthcare administrators and managers to see if UPCs remain functional at high levels or are starting to deteriorate. Early intervention can be performed in order to prevent dysfunctional UPCs.

### 4.2 Study limitations

There are some key limitations of the study. First, the study was limited to exploring the relationship between UPC functionality and nurse accountability. There was no attempt to see if such a relationship has any impact on patient outcomes, nor was there any attempt to investigate which factors affect or predict both variables. Second, the study was performed in only one tertiary hospital, thereby limiting the external validity and generalizability of results. While results suggested that UPC functionality and nurse accountability have a significant positive moderately strong relationship, the same observation may not be found in other hospitals. It may even be possible that such a result was observed because UPCs are well‐established in the research setting. It would be interesting to see levels of nurse accountability in other healthcare organizations with and without existing UPCs.

## 5 CONCLUSION

The findings of the study demonstrated moderate levels of UPC functionality and high levels of nurse accountability, though nurses scored low on recalling when the last UPC meeting was held, enumerating UPC initiatives that have been implemented in their workplaces, and holding colleagues accountable for their actions. Nurses working with highly functional UPCs have high levels of nurse accountability. Many organizations have applied some principles of shared governance, but the reach to full potential depends on many personal and organizational factors. Nursing leaders need to continuously develop strategies to empower nurses and reinforce their sense of responsibility and accountability to be an essential element in healthcare systems.

## AUTHOR CONTRIBUTIONS

MAD and HK: Planned and designed the study. MAD and MJ carried out the data collection. MAD, HK and AR contributed to the interpretation of the results. All authors discussed the results and contributed to the final manuscript.

## FUNDING INFORMATION

None to declare.

## CONFLICT OF INTEREST STATEMENT

The authors have no conflicts of interest to declare that are relevant to the content of this article.

## RESEARCH ETHICS COMMITTEE APPROVAL

Ethical approval from Zarqa Universty has been obtained (#12/2021).

## Data Availability

The data that support the findings of this study are available on request from the corresponding author. The data are not publicly available due to privacy or ethical restrictions.
